# Predictive models of immune microenvironment-related markers in patients with sepsis accompanied by myocardial dysfunction and their roles in diagnosis

**DOI:** 10.3389/fcvm.2025.1705594

**Published:** 2025-12-16

**Authors:** Xiao Zhu, Qing Lu, Xin Liu, Xianxiang Zeng

**Affiliations:** 1 Department of Intensive Care Unit, The First Hospital of Hunan University of Chinese Medicine, Changsha, Hunan, China; 2 Department of Intensive Care Medicine, The First Affiliated Hospital of Changsha Medical University, Changsha, Hunan, China; 3 Department of Sleep Disorders, Hunan Second Provincial People’s Hospital (Hunan Brain Hospital), Changsha, Hunan, China

**Keywords:** sepsis, sepsis-induced myocardial dysfunction, triggering receptor expressed on myeloid cells-1, high mobility group box 1, predictive model

## Abstract

**Objective:**

To evaluate immune microenvironment markers for predicting sepsis-induced myocardial dysfunction (SIMD) and establish three predictive models—nomogram, decision tree, and gradient boosting machine (GBM)—to compare their efficacy in assessing SIMD risk.

**Method:**

A retrospective analysis was conducted on the clinical data of 165 patients with sepsis who were admitted between January 2022 and February 2025. Patients were divided into SIMD and non-SIMD groups according to the occurrence of SIMD. Risk factors influencing the occurrence of SIMD in patients with sepsis were screened using univariate and multivariate logistic regression analyses. Nomogram, decision tree, and GBM models were constructed based on the results of the multivariate logistic regression analysis. The area under the receiver operating characteristic curve (AUC) was used to evaluate the discrimination of each model. The accuracy, sensitivity, specificity, and F1 scores of the three models were calculated.

**Result:**

: Among the 165 patients with sepsis included in the study, 75 were in the SIMD group, accounting for 45.45% (75/165). Univariate analysis showed significant differences between the two groups in APACHE II score, white blood cell count, N-terminal pro-brain natriuretic peptide (NT-proBNP), soluble triggering receptor expressed on myeloid cells-1 (sTREM-1), and high mobility group box 1 (HMGB1) levels (*P* < 0.05). Logistic regression analysis revealed that a high APACHE II score (OR = 1.480, 95% CI: 1.127–1.945), high NT-proBNP level (OR = 1.013, 95% CI: 1.005–1.021), high sTREM-1 level (OR = 1.116, 95% CI: 1.034–1.205), and high HMGB1 level (OR = 1.006, 95% CI: 1.002–1.011) were risk factors for SIMD in patients with sepsis (*P* < 0.05). All three prediction models demonstrated excellent performance in the training set: nomogram (AUC = 0.843), decision tree (AUC = 0.815), and GBM (AUC = 0.885). No significant differences were observed in the AUC values among the models (all *P* > 0.05).

**Conclusion:**

The immune markers, sTREM-1 and HMGB1, were associated with SIMD. Elevated APACHE II score and NT-proBNP, sTREM-1, and HMGB1 levels are risk factors for SIMD in patients with sepsis. Predictive models based on these factors demonstrate strong performance and effectively identify high-risk individuals, aiding in early clinical intervention.

## Introduction

Sepsis is a common disease in the field of emergency and critical care that can cause damage to organs such as the heart and liver, with the heart being one of the most frequently affected organs (1). Sepsis-induced myocardial dysfunction (SIMD) is an important component of organ dysfunction caused by sepsis with an incidence rate as high as 70% (2). The occurrence of SIMD significantly increased patient mortality. SIMD, also known as sepsis-induced cardiomyopathy (SIC), is characterized by reversible myocardial dysfunction (3). Currently, there is no gold standard or consensus for the diagnosis of SIMD. Referring to the diagnostic criteria of previous studies, diagnosis mainly relies on echocardiographic detection of abnormal ventricular function and elevated biomarkers, such as troponin and brain natriuretic peptide (4). However, these indicators lack specificity and often change only after myocardial injury occurs, limiting their early warning value. Therefore, identifying reliable indicators for early recognition of sepsis complicated by SIMD is an important topic in critical care research.

In recent years, the core role of immune mechanisms in the pathophysiology of sepsis has become increasingly evident. The severe immune disorder that occurs in the early stage of sepsis not only involves excessive inflammatory responses (5) but is also accompanied by a persistent immunosuppressive state (6). This process involves the dysfunction of multiple immune cells and release of large amounts of inflammatory and inhibitory mediators. Among these, markers such as soluble triggering receptor expressed on myeloid cells-1 (sTREM-1) (7) and high mobility group box protein 1 (HMGB1) (8) play key roles in inflammatory responses, induction of apoptosis, and maintenance of immune tolerance. They not only participate in uncontrolled systemic inflammation but also act directly on myocardial cells, inhibiting contractile function and disrupting energy metabolism, thereby leading to the development of SIMD.

Therefore, detection of immune microenvironment-related markers, such as sTREM-1 and HMGB1, provides a new perspective for the early identification of high-risk patients and for achieving early risk warning at the immune mechanism level. However, research on the role of immune-related markers in predicting SIMD in sepsis patients remains limited. Therefore, the present study aimed to systematically explore the relationship between immune microenvironment markers, including sTREM-1 and HMGB1, and the occurrence of SIMD. Combined with traditional risk factors, three predictive models—nomogram, decision tree, and gradient boosting machine—were constructed and compared in terms of their efficacy, with the goal of developing algorithmic tools with higher predictive performance to provide a theoretical basis and practical framework for the early identification and risk stratification of SIMD.

## Materials and methods

### General information

A retrospective analysis was conducted on the clinical data of 165 patients with sepsis admitted to the Intensive Care Unit of our hospital between January 2022 and February 2025. Among them, there were 97 males and 68 were female. Their ages ranged from 30 to 88 years, with an average of (62.22 ± 11.92) years. The primary infection foci causing sepsis were as follows: 76 cases in the lungs, 35 cases in the abdominal cavity, 20 cases involving the whole body, 14 cases in the urinary system, and 20 cases at other sites.

The inclusion criteria were as follows: (1) age ≥18 years; (2) diagnosis of sepsis based on the “International Consensus on Sepsis and Septic Shock, 3rd Edition” (9) released in 2016; (3) Diagnosis of SIMD meeting the criteria for sepsis (10); (4) complete clinical data.

The exclusion criteria were as follows: (1) discharge within 24 h of admission or death; (2) pregnancy or lactation; (3) recent electrical cardioversion or cardiopulmonary resuscitation (within one week); and (4) presence of autoimmune diseases or malignant tumors.

This study complied with medical ethics standards and was approved by the hospital's ethics committee.

### Sample size calculation

The sample size was calculated based on the number of Events Per Variable (EPV). According to the one-tenth rule, 10 samples were required for each included independent variable (EPV = 10). After reviewing the literature, it was estimated that the incidence of SIMD in the study population was 45%. Five variables were included in the multivariate regression model. According to the formula: sample size = number of included variables × EPV/incidence rate, the sample size was calculated as 5 × 10/45% = 111. A total of 165 patients with sepsis were included in the study based on the actual conditions of sepsis patients admitted to our hospital.

### Diagnosis and grouping of myocardial dysfunction

Two physicians with a title of attending physician or above, specializing in relevant fields, diagnosed myocardial dysfunction according to the criteria of Carbone et al. (10). This study focused only on cases of decreased left ventricular systolic function: patients with sepsis who exhibited a left ventricular ejection fraction (LVEF) of less than 50% upon examination 24 h after admission. Among the 165 patients with sepsis, 75 had myocardial dysfunction and were classified into the disorder group (SIMD group, 75 cases), whereas the remaining 90 were classified into the non-disorder group (non-SIMD group, 90 cases).

### Data collection

Clinical information was collected using the hospital's electronic information system. General information included age, sex, body mass index (BMI), underlying diseases (hypertension, diabetes, coronary heart disease), site of primary infection, mean arterial pressure (MAP) at admission, and heart rate (HR). The Acute Physiology and Chronic Health Evaluation II (APACHE II) scores were also recorded. Laboratory test results included white blood cell count (WBC), C-reactive protein (CRP), procalcitonin (PCT), N-terminal pro-brain natriuretic peptide (NT-proBNP), interleukin-6 (IL-6), IL-10, tumor necrosis factor-α (TNF-α), sTREM-1, and HMGB1.

Peripheral venous blood was collected from the patients before treatment. The supernatant was obtained by centrifugation, and serum levels of NT-proBNP, HMGB1, and sTREM-1 were measured using enzyme-linked immunosorbent assay (ELISA). All kits were purchased from Shanghai Enzyme-Linked Biotechnology Co., Ltd. (NT-proBNP kit: ML061452; HMGB1 kit: ML003171; and sTREM-1 kit: ML105186), and all operations were performed strictly according to the manufacturer's instructions. The intra- and inter-assay coefficients of variation for the reagent kits used were less than 10%, ensuring the reliability and accuracy of the test results.

### Model construction

1. Establishment of the logistic regression model: The clinical characteristics of patients in the SIMD and non-SIMD groups were compared using univariate analysis. Variables showing significant differences in the univariate analysis were included as independent variables and SIMD occurrence was used as the dependent variable. Logistic regression analysis was performed to identify variables associated with the occurrence of SIMD in patients with sepsis. The collinearity of the initially screened variables was assessed based on the variance inflation factor (VIF), with a VIF < 5 indicating no severe multicollinearity. Combining mathematical and clinical considerations, variables with collinearity were excluded, and the modeling variables were determined. Logistic regression analysis was used to construct the nomogram model.
2. Establishment of the decision tree model: The decision tree was constructed using the CART algorithm in the RStudio integrated development environment using the *rpart* package (version 4.1.23). The *Gini* index was used as the criterion for node splitting during the model growth. The minimum sample size for node splitting was set to 20 and the minimum sample size for the leaf nodes was set to 7. Cost complexity pruning was applied to control the model complexity and prevent overfitting. Using 10-fold cross-validation, the model error corresponding to each complexity parameter (*cp*) value was calculated. The *cp* value (*cp* = 0.012) corresponding to the simplest model, within one standard deviation of the minimum cross-validation error, was selected as the final pruning parameter.
3. Establishment of the gradient boosting machine (GBM) model: The GBM model was constructed in an RStudio environment using the *gbm* function. A two-step construction process was used. First, the initial maximum number of iterations was set to 2000, the shrinkage parameter to 0.01, and the interaction depth to 1 to restrict each tree to a single-split decision stump. The training process was monitored using 10-fold cross-validation, and the Bernoulli distribution was applied to adapt the binary outcome variables for preliminary modeling. The optimal number of iterations was determined based on the cross-validation results using the *gbm.perf(model, method* *=* *“cv”)* function. Finally, the GBM was retrained using the optimal number of iterations. After model fitting, the contribution of each predictor to SIMD was evaluated by analyzing the relative importance of the variables.

### Statistical methods

Data analysis was performed using SPSS version 23.0. Measurement data are expressed as mean ± standard deviation ($\bar{x}\pm s$), and comparisons between groups were made using the independent-samples t-test. Categorical data are expressed as counts and percentages [*n* (%)], and comparisons between groups were made using the *χ*^2^-test. Logistic regression analysis was performed to identify the influencing factors.

Seventy percent of the samples were randomly selected as the training set to construct the model, and the remaining 30% were used as the validation set to evaluate model performance. The influencing factors were imported into RStudio to construct nomogram, decision tree, and GBM predictive models. Model performance was assessed by calculating and comparing indicators including accuracy, sensitivity, specificity, F1 score, and area under the receiver operating characteristic curve (AUC). Statistical significance was set at *P* < 0.05. The DeLong test was used for pairwise AUC comparisons between the three prediction models. For both the training and validation sets, the Bonferroni method was applied to correct multiple comparisons. Because three pairwise comparisons were performed for each dataset, the corrected significance level was set at α = 0.0167.

## Results

### Comparison of general data between the training Set and the validation Set

Among 165 patients, 75 (45.45%) developed SIMD. The comparison of general data between the patients in the training set and those in the validation set showed no significant differences in age or other characteristics (*P* > 0.05), as shown in Table 1.

**Table 1:** Comparison of clinical data of patients between the training set and the validation set [*n*(%), ($\bar{x}\pm s$)**]**.

Factor	Training set (*n* = 115)	Validation set (*n* = 50)	*χ*^2^/*t*	*P*
Age (yrs)	62.05 ± 12.03	62.60 ± 11.78	0.271	0.787
BMI (kg/m^2^)	23.19 ± 1.74	23.13 ± 1.75	0.184	0.854
Gender				
Female	46 (40.00)	22 (44.00)	0.230	0.631
Male	69 (60.00)	28 (56.00)		
Primary infection focus				
Lung	50 (43.48)	26 (52.00)	2.713	0.616
Abdominal cavity	26 (22.61)	9 (18.00)		
The whole body	14 (12.17)	6 (12.00)		
Urinary system	12 (10.43)	2 (4.00)		
Others	13 (11.30)	7 (14.00)		
Hypertension				
No	75 (65.22)	32 (64.00)	0.023	0.880
Yes	40 (34.78)	18 (36.00)		
Diabetes				
No	95 (82.61)	43 (86.00)	0.293	0.588
Yes	20 (17.39)	7 (14.00)		
Coronary heart disease				
No	85 (73.91)	41 (82.00)	1.263	0.261
Yes	30 (26.09)	9 (18.00)		
MAP (mmHg)	82.77 ± 8.91	81.48 ± 8.51	0.869	0.386
HR (beats/min)	117.26 ± 9.68	116.40 ± 10.20	0.517	0.606
APACHEI Ⅱ score	19.60 ± 1.81	19.72 ± 2.02	0.378	0.706
White blood cell count (×10^9^/L)	11.33 ± 2.14	11.38 ± 1.68	0.160	0.873
CRP (mg/L)	122.10 ± 13.21	121.74 ± 15.7	0.154	0.878
PCT (ng/mL)	1.21 ± 0.25	1.18 ± 0.24	0.664	0.507
NT-proBNP (pg/mL)	525.79 ± 66.62	527.54 ± 55.84	0.162	0.871
IL-6 (pg/mL)	48.44 ± 9.79	48.40 ± 9.83	0.026	0.979
IL-10 (pg/mL)	18.57 ± 4.76	19.42 ± 4.69	1.064	0.289
TNF-α (pg/mL)	24.29 ± 5.86	22.70 ± 6.23	1.570	0.118
sTREM-1 (pg/mL)	36.74 ± 6.79	38.466 ± 6.05	1.544	0.124
HMGB1 (ng/mL)	1,025.03 ± 114.27	1,036.32 ± 94.25	0.614	0.540

*N*MAP, Mean arterial pressure; HR, Heart rate; APACHE II Score, Acute Physiology and Chronic Health Evaluation II Score; CRP, C-Reactive protein; PCT, Procalcitonin; NT-proBNP, N-Terminal Pro-B-Type natriuretic peptide; IL-6, Interleukin-6; IL-10, Interleukin-10; TNF-α, tumor necrosis factor-α; sTREM-1, soluble tremen-1; HMGB1, high mobility group box protein B1.

### Comparison of general information of patients in the training Set

The comparison of general data among the 115 patients in the training set showed significant differences in the APACHE II score, white blood cell count, NT-proBNP, sTREM-1, and HMGB1 levels (*P* < 0.05), as shown in Table 2.

**Table 2:** Comparison of clinical data of patients in the training Set [*n*(%), ($\bar{x}\pm s$)**]**.

Factor	Non-SIMD group (*n* = 60)	SIMD group (*n* = 55)	χ^2^/*t*	*P*
Age (yrs)	63.50 ± 12.44	60.47 ± 11.47	1.353	0.179
BMI (kg/m^2^)	23.16 ± 1.71	23.22 ± 1.80	0.209	0.835
Gender				
Female	25 (41.67)	21 (38.18)	0.145	0.703
Male	35 (58.33)	34 (61.82)		
Primary infection focus				
Lung	26 (43.33)	24 (43.64)	1.562	0.816
Abdominal cavity	13 (21.67)	13 (23.64)		
The whole body	6 (10.00)	8 (14.55)		
Urinary system	8 (13.33)	4 (7.27)		
Others	7 (11.67)	6 (10.91)		
Hypertension				
No	40 (66.67)	35 (63.64)	0.116	0.733
Yes	20 (33.33)	20 (36.36)		
Diabetes				
No	49 (81.67)	46 (83.64)	0.077	0.781
Yes	11 (18.33)	9 (16.36)		
Coronary heart disease				
No	47 (78.33)	38 (69.09)	1.271	0.260
Yes	13 (21.67)	17 (30.91)		
MAP (mmHg)	83.73 ± 8.25	81.73 ± 9.54	1.209	0.229
HR (beats/min)	116.68 ± 9.32	117.89 ± 10.09	0.667	0.506
APACHEI Ⅱ score	19.15 ± 1.77	20.09 ± 1.72	2.880	0.005
White blood cell count (×10^9^/L)	10.88 ± 1.87	11.82 ± 2.31	2.394	0.018
CRP (mg/L)	122.13 ± 14.12	122.07 ± 12.26	0.024	0.981
PCT (ng/mL)	1.24 ± 0.24	1.18 ± 0.26	1.211	0.228
NT-proBNP (pg/mL)	500.87 ± 59.65	552.98 ± 63.56	4.536	<0.001
IL-6 (pg/mL)	46.97 ± 9.98	50.05 ± 9.41	1.704	0.091
IL-10 (pg/mL)	18.65 ± 5.33	18.47 ± 4.11	0.199	0.843
TNF-α (pg/mL)	23.62 ± 6.40	25.02 ± 5.15	1.286	0.201
sTREM-1 (pg/mL)	34.27 ± 6.54	39.44 ± 6.03	4.393	<0.001
HMGB1 (ng/mL)	990.52 ± 88.76	1,062.67 ± 127.24	3.497	0.001

MAP, Mean arterial pressure; HR, Heart rate; APACHE II Score, Acute Physiology and Chronic Health Evaluation II Score; CRP, C-Reactive protein; PCT, Procalcitonin; NT-proBNP, N-Terminal Pro-B-Type natriuretic peptide; IL-6, Interleukin-6; IL-10, Interleukin-10; TNF-α, tumor necrosis factor-α; sTREM-1, soluble tremen-1; HMGB1, high mobility group box protein B1.

### Logistic regression analysis of SIMD in patients

Indicators with significant differences in the univariate analysis (APACHE II score, white blood cell count, NT-proBNP, sTREM-1, and HMGB1 levels) were taken as independent variables, and the occurrence of SIMD was taken as the dependent variable (yes = 1, no = 0). Multicollinearity diagnosis showed that the variance inflation factor (VIF) of all variables was much less than five, indicating no severe multicollinearity among the variables, making them suitable for inclusion in the multivariate regression model for further analysis (Table 3).

**Table 3:** Multicollinearity diagnosis results of each variable.

Variable	Collinearity statistics	
	Tolerance	VIF
APACHEI Ⅱ score	0.995	1.005
WBC	0.937	1.068
NT-proBNP	0.916	1.091
sTREM-1	0.873	1.146
HMGB1	0.948	1.055

APACHE II score, Acute Physiology and Chronic Health Evaluation II score; WBC, white blood cell count; NT-proBNP, N-terminal pro-brain natriuretic peptide; sTREM-1, soluble tremen-1; HMGB1, high-mobility group box protein 1.

The assignment of each variable used in the logistic regression analysis is presented in Table 4. The results of the Hosmer–Lemeshow (H–L) test showed *χ*^2^ = 4.842 and *P* = 0.774, suggesting that there was no overfitting in the model. Logistic regression analysis indicated that a high APACHE II score and high NT-proBNP, sTREM-1, and HMGB1 levels were risk factors for SIMD in patients with sepsis (*P* < 0.05), as shown in Table 5.

**Table 4:** Assignment of variables.

Variable	Assignment V
Dependent variable	
	0 = No SIMD occurred, 1 = SIMD occurred
Independent variable	
APACHEI Ⅱ score	Enter the actual value
WBC	Enter the actual value
NT-proBNP	Enter the actual value
sTREM-1	Enter the actual value
HMGB1	Enter the actual value

APACHE II score, Acute Physiology and Chronic Health Evaluation II score; WBC, white blood cell count; NT-proBNP, N-terminal pro-brain natriuretic peptide; sTREM-1, soluble tremen-1; HMGB1, high-mobility group box protein 1.

**Table 5:** Multivariate analysis.

Variable	*β*	SE	Wald *χ*^2^	*P*	OR	95%CI
APACHEI Ⅱ score	0.392	0.139	7.938	0.005	1.480	1.127–1.945
WBC	0.131	0.113	1.340	0.247	1.140	0.913–1.423
NT-proBNP	0.013	0.004	9.774	0.002	1.013	1.005–1.021
sTREM1	0.110	0.039	7.943	0.005	1.116	1.034–1.205
HMGB1	0.006	0.002	6.940	0.008	1.006	1.002–1.011
Constant	−26.305	5.161	25.978	–	–	–

APACHE II score, Acute Physiology and Chronic Health Evaluation II score; WBC, white blood cell count; NT-proBNP, N-terminal pro-brain natriuretic peptide; sTREM-1, soluble tremen-1; HMGB1, high mobility group box protein 1.

### Construction of the nomogram risk prediction model

**Figure 1:**
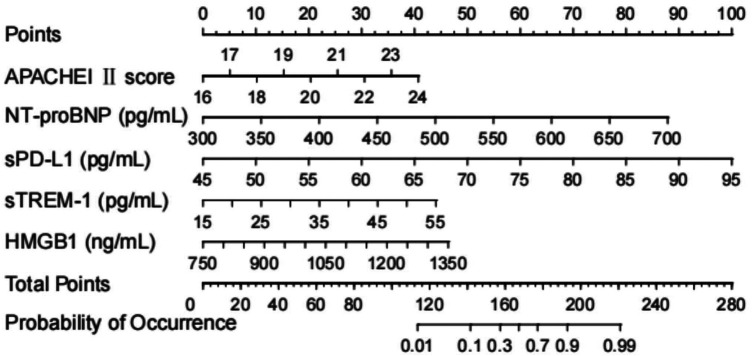
Nnomogram prediction model. APACHE II score, Acute Physiology and Chronic Health Evaluation II score; NT-proBNP, N-terminal pro-brain natriuretic peptide; sTREM-1, soluble tremen-1; HMGB1, high-mobility group box protein 1.

Based on the above logistic regression analysis, the four influencing factors were incorporated into the nomogram model for visualization; the resulting nomogram model is shown in Figure 1. The specific prediction model is as follows:

$$\begin{array}{rl} \mathrm{Logit}(P)= & -26.305+0.392\times (\mathrm{APACHE}\;\mathrm{II}\;\mathrm{score})\\ & +0.013\times (\mathrm{NT}\text{-}\mathrm{proBNP})+0.110\times (\mathrm{sTREM}\text{-}1)\\ & +0.006\times (\mathrm{HMGB}1). \end{array}$$

The nomogram model consisted of various influencing factors and corresponding line segments of specific lengths. Based on individual patient data, the scores for each influencing factor were obtained. The total score was calculated by summing the individual scores. Drawing a vertical line downward from the total score axis allows for the estimation of the patient's risk value for poor prognosis.

### Construction of the decision tree model

**Figure 2:**
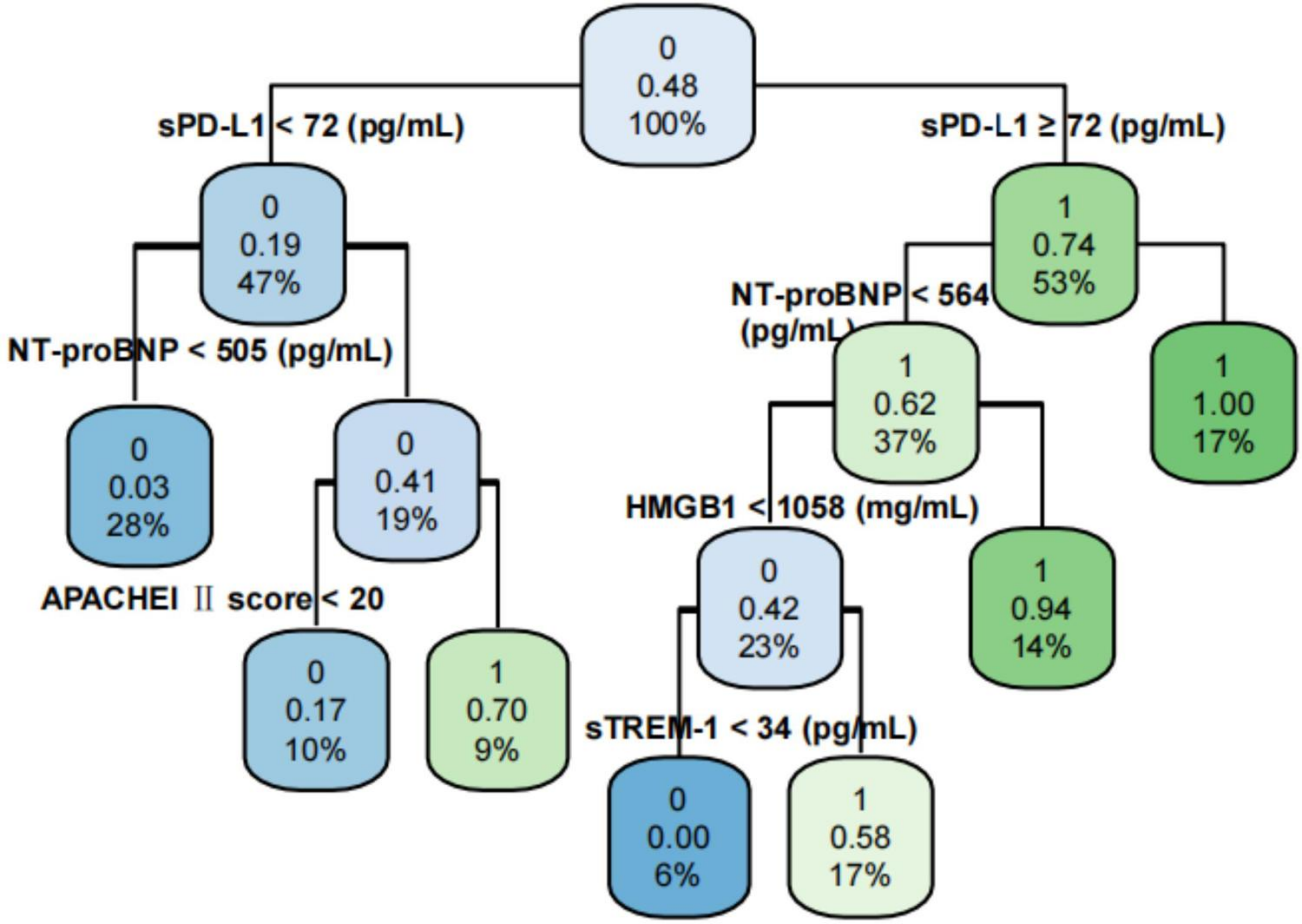
Decision tree prediction model. 0, no SIMD occurred, 1 indicates SIMD; APACHE II score, Acute Physiology and Chronic Health Evaluation II score; NT-proBNP, N-terminal pro-brain natriuretic peptide; sTREM-1, soluble tremen-1; HMGB1, high-mobility group box protein 1.

Based on the results of multivariate logistic regression analysis, a decision tree model was constructed, as shown in Figure 2. This model contains eight nodes and five decision paths. Among them, there were three decision-making paths with an SIMD occurrence risk ≥50%: ① NT-proBNP ≥ 535 pg/mL and APACHE II score ≥ 20; ② NT-proBNP ≥ 535 pg/mL, APACHE II score < 20, and HMGB1 ≥ 1,024 ng/mL; and ③ NT-proBNP < 535 pg/mL and HMGB1 ≥ 1,121 ng/mL.

### Construction of the GBM model

**Figure 3:**
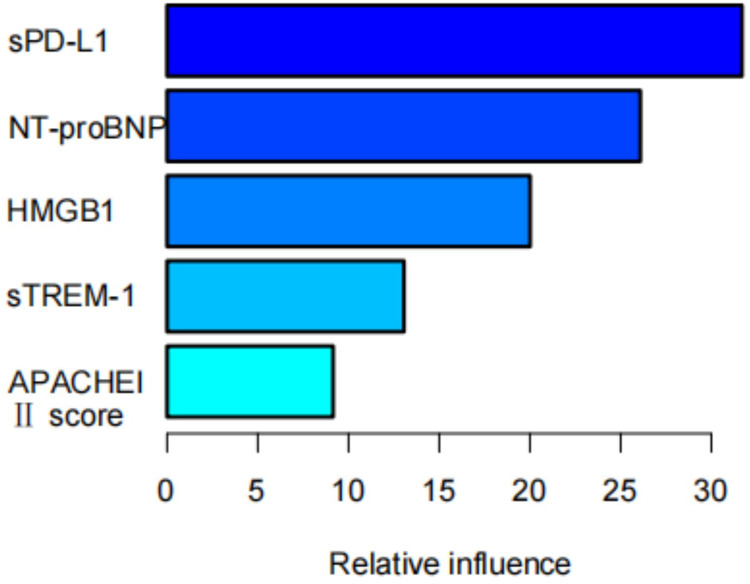
GBM prediction model. APACHE II score, Acute Physiology and Chronic Health Evaluation II score; NT-proBNP, N-terminal pro-brain natriuretic peptide; sTREM-1, soluble tremen-1; HMGB1, high-mobility group box protein 1.

The GBM model was constructed based on the results of the multivariate logistic regression analysis (Figure 3). In the GBM model, the relative importance of various clinical features obtained through the GBM algorithm, from highest to lowest, were HMGB1, NT-proBNP, sTREM-1, and APACHE II scores.

### Evaluation and comparison of the performance of the three models

**Figure 4:**
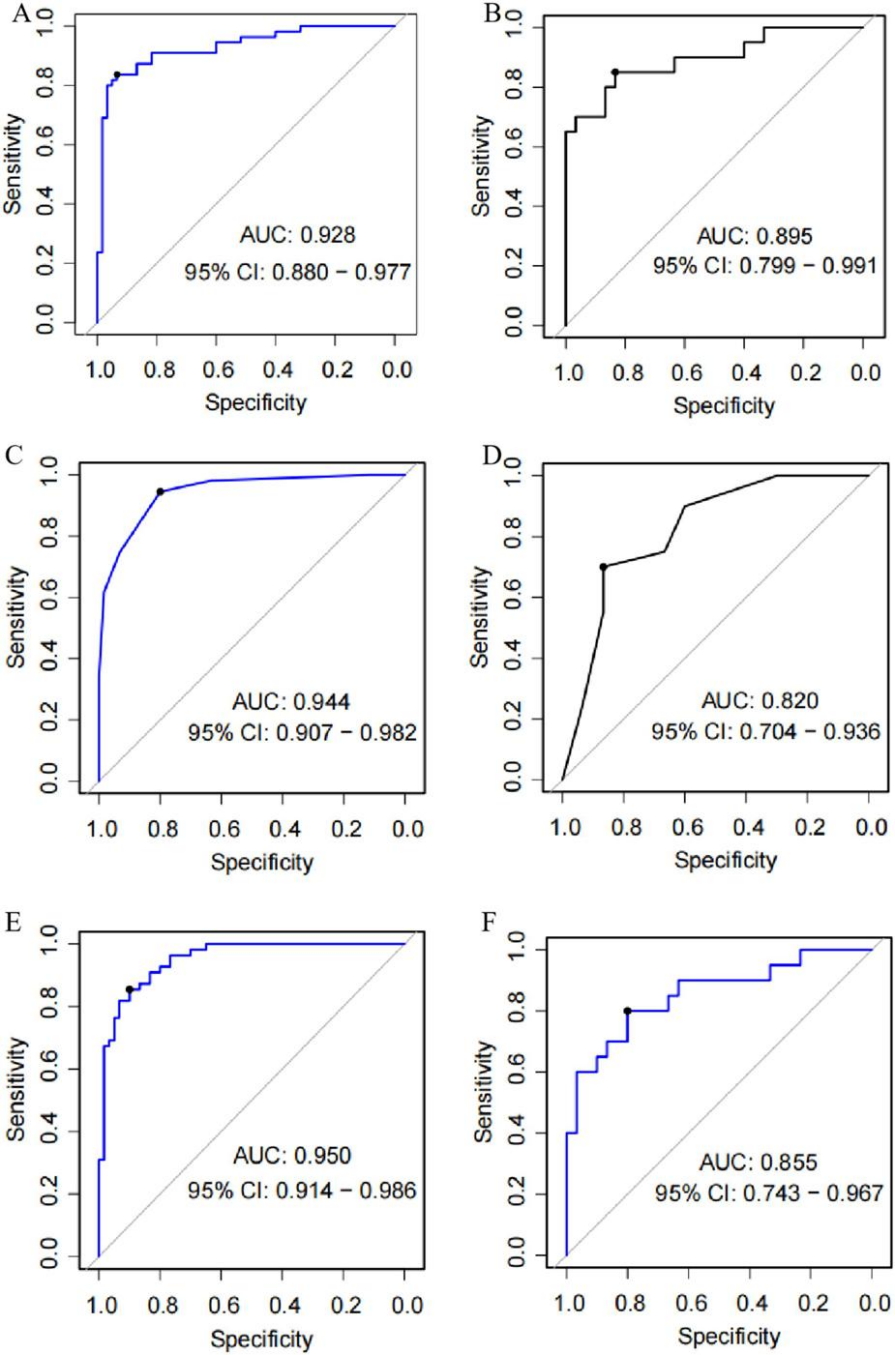
ROC test of three prediction models. **(A)** training set of nomogram model, **(B)** validation set of nomogram model, **(C)** training set of decision tree model, **(D)** validation set of decision tree model, **(E)** training set of gradient boosting machine model, **(F)** validation set of gradient boosting machine model.

The discriminatory performance of each model was assessed using ROC curves for both the training and validation sets. As shown in Figure 4. 1) in the nomogram model, the AUC of the training set was 0.843 (95% CI: 0.771–0.914), and that of the validation set was 0.812 (95% CI: 0.687–0.937); 2) in the decision tree model, the AUC of the training set was 0.815 (95% CI: 0.738–0.892), and that of the validation set was 0.658 (95% CI: 0.510–0.807); and 3) in the GBM model, the AUC of the training set was 0.885 (95% CI: 0.827–0.943), and that of the validation set was 0.790 (95% CI: 0.665–0.915).

Comparing the predictive performance of the three models showed no significant differences in AUC values between the models in either the training or validation sets (all *P* > 0.05), as shown in Table 6.

**Table 6:** AUC comparison of three prediction models.

Models	Dataset	Original *P* value	The corrected *P* value	Significance
Nomogram VS Decision tree	Training set	0.480	1.000	Not significant
Nomogram VS GBM	Training set	0.423	1.000	Not significant
Decision tree VS GBM	Training set	0.187	0.560	Not significant
Nomogram VS Decision tree	Validation set	0.125	0.374	Not significant
Nomogram VS GBM	Validation set	0.421	1.000	Not significant
Decision tree VS GBM	Validation set	0.193	0.578	Not significant

When comparing accuracy, sensitivity, specificity, and F1 values across models: 1) in the training set, the accuracy ranking was decision tree > GBM > nomogram; the sensitivity ranking was GBM = decision tree > nomogram; the specificity ranking was decision tree > GBM > nomogram; and the F1 value ranking was decision tree > GBM > nomogram; 2) in the validation set, the accuracy ranking was GBM > nomogram > decision tree; the sensitivity ranking was decision tree > GBM > nomogram; the specificity ranking was nomogram > GBM > decision tree; and the F1 value ranking was GBM > decision tree > nomogram. Detailed data are presented in Table 7.

**Table 7:** Comparison of accuracy among three prediction models.

Models	Accuracy (%)		Sensitivity (%)		Specificity (%)		F1 value	
	Training set	Validation set	Training set	Validation set	Training set	Validation set	Training set	Validation set
Nomogram	0.756	0.700	0.833	0.600	0.673	0.850	0.781	0.706
Decision tree	0.809	0.680	0.850	0.733	0.764	0.600	0.823	0.733
GBM	0.774	0.720	0.850	0.667	0.691	0.800	0.797	0.741

## Discussion

With the continuous iteration and update of sepsis guidelines, sepsis has entered the era of 3.0, which emphasizes organ dysfunction as the core and defines multiple organ dysfunction syndrome through sequential organ failure (11). SIMD plays a key role in the dysfunction of multiple organs during sepsis. Once this occurs, the mortality rate increases significantly (12). It has been 40 years since Parker et al. (13) first reported SIMD; however, the pathological mechanism has not yet been fully elucidated. Multiple pathological pathways and numerous factors are involved in its occurrence and progression. The diagnostic criteria have not yet reached consensus, making SIMD a major focus of attention in global critical care medicine. Therefore, identifying the risk factors influencing SIMD and its clinical characteristics is urgently needed to facilitate early prediction. The latest study by Sarıdaş et al. (14) developed the CALLY index, which integrates inflammation (CRP), nutrition (albumin), and immune (lymphocyte) status, and confirmed that it has a good predictive value for sepsis mortality.

The results of this study showed that a high APACHE II score was an independent risk factor for SIMD (OR = 1.554, 95% CI: 1.101–2.193). The APACHE II score, an indicator used to evaluate disease severity in critically ill patients, reflects the degree of systemic organ dysfunction as it increases (15). Huang et al. indicated that septic patients with higher APACHE II scores are more prone to developing SIMD, which is consistent with our findings (16). NT-proBNP is a neuroendocrine hormone synthesized and secreted by ventricular myocytes in response to heart failure, and is commonly used as a marker of ventricular function (17). Studies have shown that in sepsis-induced heart failure, NT-proBNP secretion increases significantly, which may be related to proinflammatory factors, mechanical ventilation, and the use of vasoactive drugs (18). As a clinical diagnostic marker, NT-proBNP has a high predictive value for the prognosis of sepsis complicated with pulmonary, intestinal, and urinary tract infections (19). In this study, the NT-proBNP level in the SIMD group was significantly higher than that in the non-SIMD group and was an independent risk factor in the multivariate analysis (OR = 1.019, 95% CI: 1.008–1.030). He et al. (20) also indicated that NT-proBNP is an independent risk factor for SIMD, exhibiting moderate diagnostic accuracy (area under the summary curve: 0.810), which is consistent with our findings. Elevated NT-proBNP levels may result from ventricular dilation and increased wall tension induced by sepsis as well as direct stimulation of myocardial cells by inflammatory cytokines to synthesize and release BNP (5).

This study also found that elevated levels of sTREM-1 and HMGB1 were independent risk factors for SIMD, and that these markers reflect the disordered immune microenvironment in sepsis. The sTREM-1 level in the SIMD group was significantly higher than that in the non-SIMD group, and it remained an independent risk factor in multivariate analysis (OR = 1.116, 95% CI: 1.034–1.205). sTREM-1 can amplify inflammatory responses, promote the release of pro-inflammatory cytokines, and participate in the pathological process of sepsis (21, 22). In myocardial injury, sTREM-1 may promote inflammation and cytokine release by activating the NF-κB signaling pathway. However, excessive cytokine production can inhibit immune function, leading to immunosuppression (23). Wei et al. (24) found that sTREM-1 is a reliable indicator of organ dysfunction in sepsis, which is consistent with our findings.

HMGB1 is a chromosome-binding protein involved in regulating inflammatory responses and stabilizing the nuclear structure. It is secreted by inflammatory or necrotic cells and is released into the extracellular fluid to mediate inflammation. HMGB1 is highly expressed in patients with sepsis and acute kidney injury (25, 26). In this study, the HMGB1 level in the SIMD group was significantly higher than that in the non-SIMD group, and it was an independent risk factor in multivariate analysis (OR = 1.006, 95% CI: 1.002–1.011). HMGB1 activates downstream signaling pathways by binding to receptors, such as TLR4 and RAGE, inducing the release of inflammatory factors and apoptosis (27, 28). During myocardial injury, HMGB1 may contribute to cardiac dysfunction through inflammatory responses and direct myocardial toxicity. Zhang et al. (29) found that HMGB1 was involved in the pathological process of myocardial injury in sepsis. Anti-HMGB1 treatment alleviated myocardial injury and improved cardiac function, consistent with our findings.

In addition, we constructed and compared three predictive models: nomogram, decision tree, and GBM. There were no significant differences in the AUC values among the models in either the training or validation sets (all *P* > 0.05). In terms of clinical practicality, the nomogram provides an intuitive risk assessment tool that allows clinicians to rapidly calculate the SIMD risk based on patient scores for various indicators, making it convenient for clinical use. The decision tree model provides clear classification rules; for example, the SIMD risk is high when “NT-proBNP ≥ 535 pg/mL and APACHE II score ≥ 20.” Such rules are easy for clinicians to understand, verify, and apply directly to clinical decision-support systems. In this study, the GBM model achieved the highest AUC (0.885) in the training set, demonstrating a strong predictive capability and pattern recognition potential. As a complex integrated algorithm, GBM can capture intricate nonlinear relationships and interaction effects between predictor variables and outcomes, which may explain its superior performance. However, its decision-making process is not intuitively interpretable and usually requires indirect methods such as ranking variable importance to reveal its internal logic. Therefore, the GBM is more suitable as a high-precision background screening tool or as an aid for senior clinicians in complex case analyses in large medical centers. In clinical applications, appropriate models can be selected based on specific requirements.

This study has several limitations. First, it was a single-center retrospective study, which may have introduced selection bias. Second, the sample size is relatively small. Third, only biomarker levels at admission were measured without dynamic monitoring. Fourth, novel biomarkers were not identified in this study. Future studies should conduct multicenter prospective validations to confirm the clinical applicability of these models, enhance dynamic biomarker monitoring to explore temporal changes, and integrate multi-omics techniques to discover new predictive markers.

In conclusion, this study confirmed that the APACHE II score, NT-proBNP, sTREM-1, and HMGB1 were independent risk factors for SIMD in patients with sepsis. The three predictive models—nomogram, decision tree, and GBM—constructed based on these indicators, all demonstrated excellent predictive performance. These models are valuable for the early identification of high-risk patients with SIMD and provide a theoretical basis for timely clinical interventions.

## Data Availability

The original contributions presented in the study are included in the article/Supplementary Material, further inquiries can be directed to the corresponding authors.
